# Flowers and Inflorescences of Selected Medicinal Plants as a Source of Triterpenoids and Phytosterols

**DOI:** 10.3390/plants12091838

**Published:** 2023-04-29

**Authors:** Pauline Edorh Tossa, Morgan Belorgey, Soyol Dashbaldan, Cezary Pączkowski, Anna Szakiel

**Affiliations:** 1 Clermont Auvergne Institut National Polytechnique, SIGMA Clermont, Campus des Cézeaux CS 20265, 63178 Aubière, France; p.edorhtossa@gmail.com; 2 Faculté de Pharmacie, Université Clermont Auvergne, 28 Place Henri Dunant, BP 38, 63001 Clermont-Ferrand, France; morgan.belorgey@hotmail.com; 3 School of Industrial Technology, Mongolian University of Science and Technology, 8th Khoroo, Baga Toiruu 34, Sukhbaatar District, Ulaanbaatar 14191, Mongolia; soyol_d@must.edu.mn; 4 Department of Plant Biochemistry, Faculty of Biology, University of Warsaw, 1 Miecznikowa Street, 02-096 Warsaw, Poland; c.paczkowski@uw.edu.pl

**Keywords:** flowers, GC-MS analysis, inflorescences, medicinal plants, phytosterols, triterpenoids

## Abstract

Steroids and triterpenoids are compounds valued for their various biological and pharmacological properties; however, their content in medicinal and edible plants is often understudied. Flowers have been consumed since the ancient times as a part of traditional cuisine and as alternative medicines. Currently, the interest in medicinal and edible flowers is growing since contemporary consumers are incessantly seeking innovative natural sources of bioactive compounds. The aim of this report was the GC-MS (gas-chromatography-mass spectrometry) analysis of steroid and triterpenoid content in flowers, inflorescences and leaves of several plants (*Berberis vulgaris* L., *Crataegus laevigata* (Poir.) DC., *Pulsatilla vulgaris* Mill., *Rosa rugosa* Thunb., *Sambucus nigra* L. and *Vinca minor* L.), applied in herbal medicine in various forms, including isolated flowers (Flos), inflorescences (Inflorescentia) or aerial parts (Herba, i.e., combined flowers, leaves and stems). The most abundant source of triterpenoids was *V. minor* flowers (6.3 mg/g d.w.), whereas the steroids were prevailing in *P. vulgaris* flowers (1.8 and 1.1 mg/g). The profiles of triterpenoid acids and neutral triterpenoids in *C. laevigata* and *S. nigra* inflorescences were particularly diverse, involving compounds belonging to lupane-, oleanane- and ursane-type skeletons. The obtained results revealed that some flowers can constitute an abundant source of phytosterols and bioactive triterpenoids, valuable for utilization in functional foods, dietary supplements and cosmetic products.

## 1. Introduction

Flowers have been consumed since the ancient times as a part of traditional cuisine and as alternative medicines. Flowers are reproductive organs of angiosperms; therefore, flowering plants usually accumulate high levels of defense metabolites in flower tissues, investing more in constitutive resistance rather than inducible resistance, because of the high value of flowers and predictability of attack of herbivores. Flowers are plant resource sinks, often containing several times more nutritional and defense compounds than other tissues, e.g., *Brassica nigra* inflorescences contained 1.2 to 4 times higher concentrations of primary metabolites than leaves, and up to 7 times higher concentrations of defense glucosinolates [1]. Therefore, flowers can be regarded as particularly valuable sources of nutritional and bioactive compounds.

Flowers have been an integral part of human culture since ancient times; they have been used for their therapeutic properties including anticancer, antidiabetic, anti-inflammatory, anthelmintic, immunomodulatory and antimicrobial activities for thousands of years [2,3]. Some of them have also been valued for their special taste and flavor, and used for culinary purposes, improving the aesthetic value and appearance of dishes [4,5]. Currently, the interest in medicinal and edible flowers is growing since contemporary consumers are incessantly seeking innovative natural sources of bioactive compounds [6,7]. Thus, flowers represent an important segment to expand alternative medicine, food and cosmetic markets due to their suitable sensory and nutritional characteristics, as well as the presence of bioactive compounds beneficial to human health [8,9]. Flowers are sources of natural moisturizers, flavorings and pigments, which make them very interesting to enrich modern cosmetic products, particularly skin cosmetic applications [10,11,12]. Flowers have been used in cosmetic products since ancient times, and contemporary consumers’ demand for utilizing natural botanical sources in cosmetics has grown substantially in recent years due to fewer side effects, high efficiency and easy availability [13,14].

Generally, 70–95% of flower composition is represented by water, and the major constituents of the remaining dry matter are carbohydrates, proteins, lipids, fiber, vitamins (notably A, C, E), organic acids, minerals (especially P, K, Ca and Mg) and various phytochemicals, e.g., phenolics (flavonoids, anthocyanins), carotenoids, terpenoids [2,3,9,15], occasionally also alkaloids, nitrogen-containing compounds and organosulfur compounds [16,17,18]. Flowers are recognized as particularly rich resources of volatile compounds, including mono- and sesquiterpenes; meanwhile, the data concerning the occurrence of triterpenoids and phytosterols are less available, although these compounds can significantly contribute to pharmacological and health-supporting activities, and their content in flowers can be valuable for utilization in functional foods, dietary supplements and cosmetic products. Phytosterols play various roles in promoting human health, e.g., they reduce lipid and cholesterol plasma levels and improve insulin resistance and lipid metabolism [19,20,21]. Triterpenoids are known as anti-inflammatory, antimicrobial, antiviral, hepatoprotective, antidiabetic and anticarcinogenic agents [22,23,24].

Triterpenoids seem to be of particular importance to flowers according to their presumable anti-herbivory defense properties, including insect antifeedant effects [25]. The studies on *Osmanthus* fragrans Loureiro Fl. Cochinch. revealed that the genes involved in the triterpenoid biosynthetic pathway, e.g., squalene synthase (SQS), squalene epoxidase (SQE) and beta-amyrin synthase (BETA-AS) had a clear flower-specific expression pattern, and might result in the specific accumulation of triterpenoids in this plant part [26]. Some flowers are known and appreciated sources of bioactive triterpenoids, e.g., *Chrysanthemum morifolium* Ramat (Japanese name: Ryourigiku), widely cultivated in the northeastern part of the Honshu Island as a traditional edible flower (capitulum inflorescence). The study on methanol and hexane extracts of this inflorescence demonstrated the occurrence of twenty-four triterpene diols and triols (including faradiol and heliantriol C), exerting marked anti-inflammatory activity [27]. Bioactive triterpenoid acids, e.g., oleanolic and triterpenoid acids, often accompanied by their polyhydroxylated derivatives (maslinic, corosolic, tormentic acids) were found in the flowers of several plant families, including Ericaceae and Lamiaceae [28,29]. Some reports demonstrated the accumulation of significant amounts of triterpenoid glycosides (saponins) in flowers, and lead to the discovery of new compounds with unusual structures, e.g., terpengustifol, exerting cytotoxic activity against A375 (malignant melanoma) cell lines, isolated from the flowers of *Elaeagnus angustifolia* L., used to treat asthma and thoracalgia in Chinese Uygur medicine [30]; as well as two new triterpenoid saponins, loniceroside D (hederagenin glycoside) and loniceroside E (oleanolic acid glycoside), isolated from flowers of *Lonicera japonica* Thunb. and applied for antiviral, anti-inflammatory and antibacterial activities [31].

The aim of the present study was to investigate the steroid and triterpenoid content of several flowers and inflorescences (*Berberis vulgaris* L., *Crataegus laevigata* (Poir.) DC., *Pulsatilla vulgaris* Mill., *Rosa rugosa* Thunb., *Sambucus nigra* L. and *Vinca minor* L.) applied in herbal medicine in various forms, including isolated flowers (Flos), inflorescences (Inflorescentia) or aerial parts (Herba, i.e., combined flowers, leaves and stems) by the GC-MS (gas-chromatography-mass spectrometry) method. Additionally, for *B. vulgaris*, *C. laevigata*, *P. vulgaris*, *R. rugosa* and *V. minor*, the comparison of the content of flowers/inflorescences and leaves was performed, whereas for *S. nigra,* the contents of the inflorescences of two cultivars, wild and ornamental *S. nigra* f. *porphyrophylla* Black lace “Eva”, were compared. Phytosterols and triterpenoids can contribute to the known pharmaceutical properties of medicinal plants, acting either directly or synergistically with other bioactive constituents; therefore, the obtained results supplement the existing phytochemical characteristics of the analyzed plant resources.

## 2. Results

### 2.1. Identification of Steroids and Triterpenoids in Obtained Extracts

Steroids and triterpenoids occurring in the fractions obtained from diethyl ether extracts of plant material (Section 4.3) were analyzed by the gas chromatography-mass spectrometry (GC-MS) method and identified according to their MS spectra (steroids and neutral triterpenoids without derivatization, triterpenoid acids after methylation). The identification was additionally supported by a comparison of their retention time and chromatographic mobility to the respective parameters of available authentic standards, as well as a comparison with data from MS libraries and the literature (Section 4.5, Table S1).

**Figure 1:**
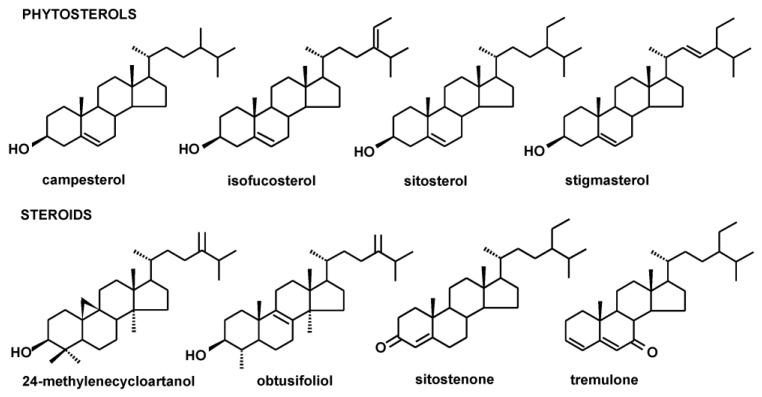
The structures of identified phytosterols and steroids.

Identified steroids comprised four typical plant sterols, belonging to the group commonly known as phytosterols, i.e., campesterol (24*R*)-ergost-5-en-3β-ol), sitosterol (stigmast-5-en-3β-ol), stigmasterol (22*E*)-stigmasta-5,22-dien-3β-ol) and isofucosterol (24Z-stigmasta-5,24(28)-dien-3β-ol, synonym: Δ5-avenasterol]. Additionally, the saturated form of sitosterol, i.e., sitostanol; two compounds considered as biosynthetic intermediates, i.e., 24-methylenecycloartanol (24-methylene-9,19-cyclolanostan-3β-ol), and obtusifoliol (24-methylene-29-nor-5α-lanost-8-en-3β-ol, synonym: 4α,14α-dimethyl-5α-ergosta-8,24(28)-dien-3β-ol) and the oxygenated derivatives of sterols (steroid ketones): sitostenone (stigmasta-4-en-3-one) and tremulone (stigmasta-3,5-dien-7-one) were also identified. The composition of steroids differed in various analyzed plants, e.g., in *C. laevigata,* extracts of only one main phytosterol, sitosterol, accompanied by its ketone, tremulone, were identified; whereas, the profiles in *P. vulgaris* and particularly *R. rugosa* were richer, comprising four phytosterols and additional steroids. The structures of the identified steroids are presented in Figure 1.

The fraction of the neutral triterpenoids, i.e., alcohols, ketones and aldehydes, comprised the most commonly occurring triterpenoid alcohols with one hydroxyl group (monols) of five types of carbon skeletons: ursane-type, i.e., α-amyrin (urs-12-en-3β-ol); oleanane-type, i.e., β-amyrin (olean-12-en-3β-ol); 18-oleanane-type, i.e., germanicol (olean-18-en-3β-ol); lupane-type, i.e., lupeol (lup-20(29)-en-33β-ol); and taraxastane-type, i.e., taraxasterol (taraxast-20(30)-en-3β-ol). Oleanane-, ursane- and lupane-type alcohols with two hydroxyl groups (diols) comprised erythrodiol (olean-12-ene-3β,28-diol), uvaol (urs-12-ene-3β,28-diol) and betulin (lup-20(29)-ene-3*α*,28-diol). One ketone, i.e., α-amyrenone, and two aldehydes, oleanolic and ursolic, were also identified. The two identified monols, α-amyrin and lupeol, and their ketones, α-amyrenone and lupenone, were associated with common peaks, as described in the previous reports [32,33]; therefore, in the extracts where lupane-type triterpenoids were present, the respective pairs of compounds were quantified together. Some triterpenoid esters, i.e., oleanolic acid acetate and methyl esters of oleanolic and ursolic acids, were identified in the fraction of the neutral triterpenoids, due to the same range of their chromatographic mobility during fractionation on silica gel plates (Rf 0.3–0.9; Section 4.3). Their calculated contents were then included in the fraction of triterpenoid acids.

**Figure 2:**
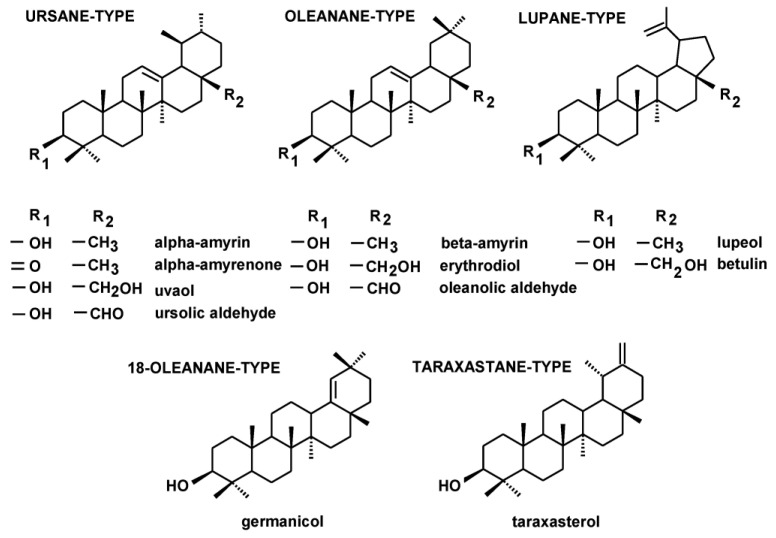
The structures of identified neutral triterpenoids.

The content and composition of neutral triterpenoids differed greatly among analyzed extracts, e.g., the complex profiles of these compounds were found in *C*. *laevigata* and *S. nigra* extracts, whereas *B. vulgaris* and *P. vulgaris* extracts did not contain any compound belonging to this group. The structures of the identified neutral triterpenoids, classified according to the skeleton type, are presented in Figure 2.

The fractions of triterpenoid acids (subjected to methylation prior to GC-MS analysis) comprised the compounds of ursane-, oleanane- and lupane-type skeletons, i.e., ursolic acid (3β-hydroxy-urs-12-en-28-oic acid), oleanolic acid (3β-hydroxy-olean-12-en-28-oic acid) and betulinic acid (3β-hydroxy-lup-20(29)-en-28-oic acid). Oleanolic and ursolic acids were accompanied by their various derivatives: 3-oxo-analogs (3-oxoolean- 12-en-28-oic acid and 3-oxours-12-en-28-oic acid) as well as analogs with an additional double bond in position two (olean-2,12-dien-28-oic acid and ursa-2,12-dien-28-oic acid). Exclusively in *R. rugosa* extracts, 2,3-dihydroxy analogs of oleanolic and ursolic acids were found, i.e., maslinic acid (2α,3β-dihydroxy-olean-12-en-28-oic acid), corosolic acid (2α,3β-dihydroxy-urs-12-en-28-oic acid) and pomolic acid (3,19-dihydroxy-urs-12-en- 28-oic acid).

**Figure 3:**
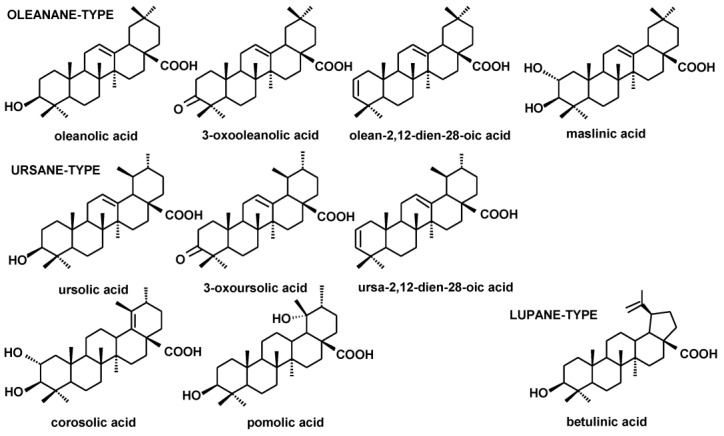
The structures of identified triterpenoid acids.

As previously in the case of the neutral triterpenoids, the obtained extracts varied markedly regarding the composition of triterpenoid acids. The profiles of *R. rugosa*, *C*. *laevigata* and *S. nigra* extracts were the most complex, whereas *P. vulgaris* and *V. minor* contained exclusively oleanolic and ursolic acids. The structures of the identified triterpenoid acids are presented in Figure 3.

### 2.2. The Content of Steroids and Triterpenoids in Berberis vulgaris Inflorescences and Leaves

The steroid profile of *B. vulgaris* inflorescences and leaves was identical, consisting of three phytosterols: campesterol, sitosterol and stigmasterol; and accompanied by 24-methylenecycloartanol and tremulone. The fraction of sterols and steroids was 3.5-fold more abundant in leaves than in inflorescences. Sitosterol was the main phytosterol, constituting 36% of the total fraction of steroids in inflorescences and 52% in leaves. The second abundant phytosterol was campesterol, followed by stigmasterol.

The neutral triterpenoids were not detected, whereas two isomeric acids, oleanolic and ursolic acids, were found both in inflorescences and leaves. However, the composition of the fraction of acids was richer in inflorescences, due to the occurrence of 3-oxo- and 2,12-dien-analogs of both acids. The fraction of acids was more abundant (by 17%) in inflorescences than in leaves. Ursolic acid was prevailing, constituting 54% and 66% of the fraction of triterpenoid acids in inflorescences and leaves, respectively.

Thus, typical features of *B. vulgaris* inflorescences and leaves were: (i) the common profile of basic phytosterols and steroids; (ii) the absence of the neutral triterpenoids, (iii) the presence of triterpenoid acids with oleanane- and ursane-type skeletons and (iv) the prevalence of steroid content. Simultaneously, several differences in the profile and content of triterpenoids and steroids were detected between the inflorescences and leaves of *B. vulgaris*, including: (i) more than two-fold prevalence of the total amount of both fractions in leaves than in inflorescences; however, with the number of triterpenoid acids slightly higher in inflorescences than in leaves and (ii) the presence of the derivatives of triterpenoid acids (oxo-analogs and the compounds with additional double bond) only in inflorescences.

The results of the quantitative analysis of compounds identified in *B. vulgaris* inflorescences and leaves are presented in Table 1.

### 2.3. The Content of Steroids and Triterpenoids in Crategus laevigata Inflorescences and Leaves

In both inflorescences and leaves, the very simple profile of sterols and steroids was detected, with only one phytosterol, sitosterol and the steroid ketone, tremulone. The content of these compounds was higher by 72% in leaves.

The fraction of the neutral triterpenoids in inflorescences was represented by α-amyrine, lupeol and β-amyrine, as well as ursolic and oleanolic aldehydes. In leaves, triterpenoid aldehydes were absent, whereas dihydroxy alcohols as erythrodiol and uvaol were detected. Additionally, germanicol and α-amyrenone were only found in leaves. In inflorescences, the sum of α-amyrine and lupeol was dominating, constituting 82% of the fraction, whereas in leaves germanicol was the most abundant (26% of the fraction). In spite of the richer profile of the neutral triterpenoids in leaves, the total content of this fraction was much more abundant (7.4-fold) in inflorescences, mainly due to the very high amount of α-amyrine and lupeol.

The fraction of triterpenoid acids was represented by ursolic, oleanolic and betulinic acids, accompanied by ursane- and oleanane-type 2,12-dien and 3-oxo-analogs (the latter present only in inflorescences). Ursolic acid prevailed both in inflorescences (60% of the fraction of acids) and leaves (75%). The total amount of fraction of acids was almost four-fold more abundant in leaves than in inflorescences.

Thus, the typical features of *C. laevigata* inflorescences and leaves were: (i) the very simple profile of sterols and steroids; (ii) the presence of triterpenoids with lupane-, oleanane- and ursane-type skeletons and (iii) the prevalence of triterpenoids over steroids.

Differences in the profile and content were: (i) more than two-fold prevalence of the total amount steroids and triterpenoids in leaves than in inflorescences; (ii) neutral triterpenoids as dominating fraction in inflorescences (54% of the total content), whereas triterpenoid acids in leaves (94%); (iii) the presence of oleanolic and ursolic aldehydes only in inflorescences, whereas the respective dihydroxy alcohols (erythrodiol and uvaol) only in leaves; (iv) the presence of germanicol only in leaves and (v) the presence of 3-oxo-analogs of oleanolic and ursolic acids only in inflorescences.

The results of the quantitative analysis of compounds identified in *C. laevigata* inflorescences and leaves are presented in Table 2.

### 2.4. The Content of Steroids and Triterpenoids in Pulsatilla vulgaris Flowers and Leaves

In both flowers and leaves, campesterol, sitosterol and stigmasterol were identified, with two steroids, sitostenone and tremulone. Another phytosterol, isofucosterol, was detected only in flowers. The fraction of steroids was 4.7-fold more abundant in flowers than in leaves. Sitosterol was the main phytosterol, constituting 57 % of the total fraction of steroids in flowers and 42% in leaves. The neutral triterpenoids were not detected. Only oleanolic and ursolic acids were found in both flowers and leaves with a predominating amount of oleanolic acid, approximately 80% of the fraction both in flowers and leaves.

The results of the quantitative analysis of compounds identified in *P. vulgaris* flowers and leaves are presented in Table 3.

Typical features of *P. vulgaris* flowers and leaves were: (i) the remarkable prevalence of the content of sterols and steroids; (ii) the absence of the neutral triterpenoids; (iii) the presence of small amounts of two basic triterpenoid acids with oleanane- and ursane-type skeletons (oleanolic and ursolic acids), with dominating oleanolic acid. Differences in the profile and content of triterpenoids and steroids were: (i) more than 4.3-fold prevalence of the total amount of triterpenoids and steroids in flowers than in leaves, (ii) the presence of isofucosterol only in flowers.

### 2.5. The Content of Steroids and Triterpenoids in Rosa rugosa Flowers and Leaves

In both flowers and leaves, four phytosterols (campesterol, isofucosterol, sitosterol and stigmasterol) were identified, with two biosynthetic intermediates, obtusifoliol and 24-methylenecycloartanol, as well as two ketones, sitostenone and tremulone. The fraction of steroids was 46% more abundant in leaves than in flowers. Sitosterol was the main phytosterol, constituting 72 % of the total fraction of steroids in flowers and 59% in leaves. The fraction of the neutral triterpenoids was represented by alcohols, aldehydes and one ketone, i.e., α-amyrine, lupeol, β-amyrine, α-amyrenone, ursolic and oleanolic aldehydes; and dihydroxy alcohols (erythrodiol and uvaol) present only in flowers. The sum of α-amyrine and lupeol was dominating, constituting 54% and 52% of the fraction in flowers and leaves, respectively. The total content of this fraction was more abundant (by 40%) in flowers.

The results of the quantitative analysis of compounds identified in *R. rugosa* flowers and leaves are presented in Table 4.

The fraction of triterpenoid acids was the most abundant and complex, consisting of basic monohydroxy acids (ursolic, oleanolic and betulinic acids) accompanied by dihydroxy analogs: oleanane-type maslinic acid, ursane-type corosolic and pomolic acids. Ursane-,oleanane-type 2,12-dien and 3-oxo-analogs were present only in leaves. Ursolic acid prevailed both in flowers (49% of the fraction of acids) and leaves (20%). The total content of the fraction of acids was comparable in flowers and leaves (only by approx. 6% higher in flowers than in leaves).

Thus, the typical features of *R. rugosa* flowers and leaves were: (i) the complex profiles of both steroids and triterpenoids; (ii) the presence of the neutral triterpenoids and acids with lupane-, oleanane- and ursane-type skeletons and (iii) the prevalence of the fraction of triterpenoid acids. Differences in the profile and content were: (i) the fraction of steroids less abundant than the fraction of the neutral triterpenoids in flowers, and the opposite in leaves; (ii) the presence of dihydroxy alcohols, erythrodiol and uvaol, exclusively in flowers and (iii) the presence of 2,12-dien and 3-oxo-analogs of oleanolic and ursolic acids only in leaves.

### 2.6. The Content of Steroids and Triterpenoids in Inflorescences of Wild S. nigra and Ornamental S. nigra f. Porphyrophylla

The results of the quantitative analysis of compounds identified as wild *S. nigra* and ornamental *S. nigra* f. *porphyrophylla* inflorescences are presented in Table 5.

The total content of all identified compounds was 32% higher in wild *S. nigra* inflorescences than in inflorescences of ornamental *S. nigra* f. *porphyrophylla* Black lace “Eva” cultivar. However, the profile of steroids and triterpenoids was identical in both inflorescences. The steroid fraction comprised three phytosterols: campesterol, sitosterol, accompanied with its saturated form, sitostanol and isofucosterol (surprisingly, stigmasterol was not detected in *S. nigra* inflorescences), the intermediate of sterol biosynthesis, 24-methylenecycloartanol and ketone tremulone. Sitosterol was the predominating among steroids (61% and 65% of the fraction in wild and ornamental inflorescences, respectively). The total content of the steroid fraction was 20% higher in wild than in ornamental inflorescences.

The fraction of the neutral triterpenoids was the less abundant, although of complex composition, consisting of monohydroxyalcohols α- and β-amyrins, germanicol, taraxasterol; dihydroxyalcohols: uvaol, erythrodiol; as well as oleanolic and ursolic aldehydes. The predominating compound was ursolic aldehyde (39% and 35% of the fraction in wild and ornamental inflorescences, respectively). Again, the fraction of the neutral triterpenoids was more abundant in wild inflorescences (by 30%).

The fraction of triterpenoid acids was composed only of oleanane- and ursane-type compounds, i.e., oleanolic and ursolic acids and their derivatives: naturally occurring methyl esters, 3-oxo- and 2,12-dien analogs. The predominating compound was ursolic acid (60% and 67% of the fraction in wild and ornamental inflorescences, respectively). The total content of triterpenoid acids was 32% higher in wild inflorescences. Generally, the triterpenoid acid fraction prevailed in both wild and ornamental inflorescences and accounted for approx. 91% of the total content of all identified compounds in both wild and ornamental *S. nigra*.

### 2.7. The Content of Steroids and Triterpenoids in Vinca minor Flowers and Leaves

In both flowers and leaves, the typical profile of phytosterols, campesterol, sitosterol and stigmasterol was identified with sitosterol as the main phytosterol in leaves (47% of the entire fraction). Among steroids, tremulone was present in both flowers and leaves, but 24-methylenecycloartanol was detected only in flowers as the prevalent compound of the fraction (38%), whereas sitostenone was only found in leaves.

The neutral triterpenoid fraction was dominating in *V. minor* extracts constituting 75% of the total content of triterpenoids and steroids in flowers and 58% in leaves. The amount of the neutral triterpenoids was 4.5-fold more abundant in flowers than in leaves. The composition of this fraction differed markedly between flowers and leaves, the only common compounds were α-amyrine, lupeol and β-amyrine. In flowers, α-amyrenone and significant amounts of lupeol acetate were detected, whereas ursolic and oleanolic aldehydes as well as betulin were found in leaves.

Despite the presence of lupane-type neutral triterpenoids, only oleanane- and ursane-type compounds were found in the fraction of acids. In leaves, exclusively oleanolic and ursolic acids were detected, whereas in flowers additionally oleanolic acid ester, acetate, was found. The amount of the fraction of acids was almost nine-fold higher in flowers than in leaves. Ursolic acid was predominating both in flowers and leaves, constituting 67% and 76% of the fraction, respectively. In flowers, the fraction of triterpenoid acids was the second most abundant (13.5% of the total triterpenoids and steroids) following the neutral triterpenoids, whereas in leaves this fraction was the less abundant (only 5%).

Typical features of *V. minor* flowers and leaves were: (i) the common profile of basic phytosterols; (ii) occurrence of neutral triterpenoids of lupane-, oleanane- and ursane- types; (iii) neutral triterpenoids as the dominating fraction (75% in flowers, 58% in leaves); (iv) the absence of lupane-type acid (betulinic acid) and (v) the presence of oleanane- and ursane-type acids, exclusively. Differences in the profile and content of triterpenoids and steroids: (i) more than 3.5-fold prevalence of the total amount of both fractions in flowers than in leaves; (ii) the presence of 24-methylenecycloartanol only in flowers, sitostenone only in leaves; (iii) the presence of α-amyrenone and two esters, lupeol acetate and oleanolic acid acetate, only in flowers, whereas betulin as well as oleanolic and ursolic aldehydes only in leaves; (iv) almost nine-fold higher abundance of triterpenoid acids in flowers than in leaves and (v) triterpenoid acids as the second abundant fraction in flowers, whereas the least abundant in leaves.

The results of the quantitative analysis of compounds identified in *V. minor* flowers and leaves are presented in Table 6.

## 3. Discussion

Plants analyzed in the present study were selected regarding their application in traditional folk and/or contemporary herbal medicine (particularly utilization based on potential anti-inflammatory, antibacterial and antiviral activities), as well as a broad availability from the licensed suppliers of crop and decorative plants, and hence their relative popularity in the gardens. The phytochemical characteristics of the selected plants have not included the detailed analysis of phytosterols and triterpenoids (apart from some general data), whereas these compounds might exert (directly or synergistically) some expected pharmacological properties ascribed to the studied herbal material. According to the criterion of diversity, the plants belonged to various taxonomic families: *B. vulgaris* to Berberidaceae, *C. laevigata* and *R. rugosa* to Rosaceae, *P. vulgaris* to Ranunculaceae, *S. nigra* to Adoxaceae and *V. minor* to Apocynaceae; they were classified as herbaceous perennials (*P. vulgaris*, *V. minor*), bushes (*B. vulgaris*, *R. rugosa*, *S. nigra*) or trees (*C. laevigata).*

The occurrence of phytochemicals belonging to different classes of metabolites (particularly those classified as specialized) varies greatly throughout the plant kingdom. The obtained results revealed that flowers and inflorescences selected for the present study differed markedly in steroid and triterpenoid composition and content. Steroids were the most abundant in *P. vulgaris* and *V. minor* flowers (0.11% and 0.08% d.w., respectively), whereas were the least abundant in *B. vulgaris* and *C. laevigata* inflorescences (0.015% and 0.012% d.w., respectively). Thus, although steroids—and particularly phytosterols—are considered general metabolites due to their architectural and regulatory role in plant membranes, the pattern of their occurrence is highly differentiated in various plant species and plant organs. In turn, the wide variability of the content of specialized triterpenoids is more predictable; indeed, in analyzed flowers/inflorescences it ranged from 1.32% to 0.89% d.w. in wild and ornamental *S. nigra* inflorescences; 0.64% in *V. minor* flowers to only approx. 0.01% in *B. vulgaris* inflorescences and 0.003% in *P. vulgaris* flowers (the values calculated for both neutral triterpenoids and acids). The richest source of the neutral triterpenoids (alcohols, ketones, aldehydes) were *V. minor* flowers (0.54% d.w.) and *C. laevigata* inflorescences (0.16% d.w.), whereas in *B. vulgaris* inflorescences and *P. vulgaris* flowers, these compounds were not detected at all. The most abundant source of triterpenoid acids was wild and ornamental *S. nigra* inflorescences (1.28% and 0.87% d.w.), followed by *C. laevigata* inflorescences (0.12% d.w.), *R. rugosa* (0.1% d.w.) and *V. minor* flowers (approx. 0.1% d.w.), whereas in *P. vulgaris* flowers these compounds were found only in trace amounts. The composition of triterpenoids was the most complex in *C. laevigata, R. rugosa, S. nigra* and *V. minor,* whereas the simplest in *P. vulgaris* (only oleanolic and ursolic acids).

It is often hypothesized that the flowers would contain higher amounts of specialized metabolites than leaves, to protect these valuable generative plant parts from herbivores and pathogens. The present study only partially confirmed that hypothesis. Indeed, in the majority of the selected species the flowers/inflorescences accumulated more triterpenoids than leaves, e.g., the content of triterpenoid acids in *V. minor* flowers was almost nine-fold higher than in leaves; and the content of the neutral triterpenoids in *C. laevigata* inflorescences was 7.4-fold higher than in leaves. However, the content of triterpenoid acids in *C. laevigata* inflorescences was almost five-fold higher in leaves than in inflorescences, finally resulting in the total content of triterpenoids being twice higher in leaves than in flowers. Therefore, the prevalence of triterpenoid accumulation in flowers should not be treated as a general rule in plant defense strategy.

In comparison to data concerning the content of other groups of compounds, particularly phenolics exerting antioxidant properties and various pigments (flavonoids, antocyanins, carotenoids) in flowers and inflorescences of angiosperms, the reports on the content of triterpenoids and steroids are still rather scarce. For instance, no detailed phytochemical characterization involving triterpenoids and phytosterols had been reported for *B. vulgaris* inflorescences, although the presence of oleanolic acid, lupeol and stigmasterol was detected in *B. vulgaris* fruit [34,35]. Oleanolic and ursolic acids, i.e., the most commonly occurring triterpenoids, were found previously in flowers and inflorescences of *C. laevigata* [36], *S. nigra* [37] and *V. minor* [38]; however, the detailed analysis of other accompanying triterpenoids and phytosterols was performed in the present study for the first time. In turn, the obtained results revealed that *P. vulgaris* flowers contained only trace amounts of these acids in a free form, which was in accordance with the recent finding that this plant appeared to be rich in triterpenoid saponins [39], i.e., triterpenoid glycosides that were not determined in the present study. On the other hand, on the basis of the previous report on the occurrence of triterpenoids in *R. rugosa* accessory fruit [40], the occurrence of significant amounts of these compounds in a free form in the flowers of this plant could be predicted.

Thus, the present results revealed that flowers and inflorescences of some medicinal plants less characterized for triterpenoid content can be valuable sources of these compounds; moreover, the data concerning the occurrence of bioactive triterpenoids in herbal material can widen the spectrum of their potential applications. According to presumable anticancer, anti-inflammatory, antidiabetic and antibacterial properties of triterpenoids, the number of new reports on the content of these compounds in various plant materials is markedly increasing in recent years, e.g., presenting the occurrence of cucurbitane-type momordicin and charantin in the flowers of white bitter melon *Momordica charantia* [41], or the complex composition of sesquiterpenoids, diterpenoids and triterpenoids in hop *Humulus lupulus* inflorescences [42]. Likewise, the investigations on the content of phytosterols, compounds valued for the regulation of cholesterol and lipid metabolism are attracting more attention, e.g., the presence of this group of compounds in various plant raw materials applied in the traditional medicine of various world regions [32,43,44,45].

Although the discovery of plant resources rich in triterpenoids and phytosterols is obviously of particular interest, the data concerning the occurrence of these compounds in lower amounts are also valuable. The synergistic action of triterpenoids with other bioactive phytoconstituents, e.g., flavonoids, has been reported recently for the antibacterial activity of plant extracts [46,47], which might be of crucial importance in the ongoing research for novel therapeutic agents against multidrug-resistant microbial strains [48]. Flavonoids are usually abundant in flowers and inflorescences, including those investigated in the present study (e.g., hyperoside and vitexin in *C. laevigata* [36], quercetine and kaempferol in *R. rugosa* [49], quercetin, isoquercitrin and anthocyanins in *S. nigra* [50]); thus, such potential synergism with triterpenoids is highly probable. Similarly, the presence of triterpenoids, exerting anti-inflammatory, antidiabetic and cardioprotective activities [22,51,52] might be significant for herbal materials known for such properties ascribed primarily to other compounds, e.g., polyphenols. Anti-inflammatory, antibacterial and antiviral activities were reported for herbal preparations from *R. rugosa* flowers [47] and *S. nigra* inflorescences [37,50], whereas *C. laevigata* inflorescences have been traditionally valued for their anti-inflammatory and cardioprotective effects [53].

Some flowers and inflorescences selected for this study (e.g., *P. vulgaris*, *V. minor*) have been applied in traditional folk medicine, as well as in contemporary herbal medicine, as a plant “aerial part”, i.e., combined flowers, leaves and stems. The obtained results revealed that the quality of such a plant material (in terms of composition and amount of bioactive phytochemicals) might be strongly dependent on the proportion among different plant parts, particularly flowers and leaves, and thus its application requires careful standardization. The chemical composition and functional properties of flowers depend on species/cultivar; in the present study, the significant differences between the wild and ornamental *S. nigra* inflorescences were demonstrated. Other important factors influencing the phytochemical content (particularly including steroid and triterpenoid profiles) of plants are the country/region of origin [32,54,55] and the external environmental conditions, including abiotic and biotic stresses [56,57].

Currently, the discovery of new edible flowers, surveys of their phytochemicals and the full utilization of known edible flowers are of great scientific and commercial interest [58]. Culinary utilization of flowers has begun with the consumption of floral parts including the stalk and flower nectar as conventional foods, gradually used as spices, and food colorants, and recently they have gained interest as functional foods and nutraceuticals [9,59]. The increased interest in potent nutraceuticals has prompted research beyond conventional plant sources (that can include rare or even endangered wild species) to cultivated ornamental plants [8,60]. Therefore, despite the growing popularity of edible flowers, it should be emphasized that there are numerous not edible plants (particularly including ornamental species), and their flowers can be even toxic and very poisonous [9,59]. For safe insertion in human food, it is necessary to correctly identify the species that produce flowers to be consumed, verify their properties and check for the presence of antinutritional, toxic and/or allergenic compounds [8,59,60,61]. Among the species selected for the present study, only *R. rugosa* flower petals could be recommended for safe consumption, the culinary application of other flowers and inflorescences is hazardous due to the presence of alkaloids, particularly *P. vulgaris* and *V. minor* flowers, despite their popularity in the gardens, as well as attractive color and appearance.

## 4. Materials and Methods

### 4.1. Plant Material

**Figure 4:**
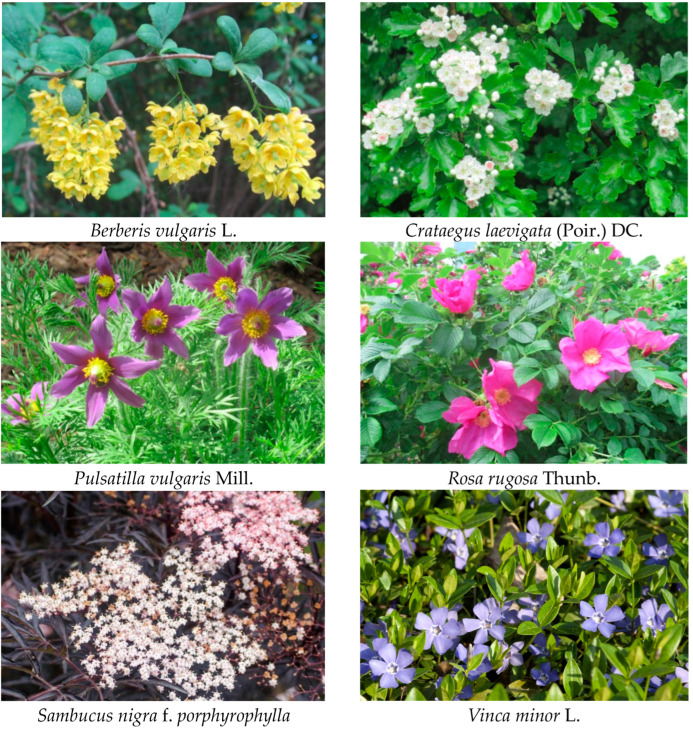
Selected plants in their flowering stage (the photographs were taken by Anna Szakiel).

Plant samples: flowers and leaves of *Berberis vulgaris* L., *Crataegus laevigata* (Poir.) DC., *Pulsatilla vulgaris* Mill., *Rosa rugosa* Thunb., *Vinca minor* L. and inflorescences of wild *Sambucus nigra* L. and ornamental *S. nigra* f. *porphyrophylla* Black lace “Eva” (Figure 4.) were collected by Anna Szakiel from a private collection of medicinal and ornamental plants in Stare Bosewo, central Poland (52°460 N, 21°332 E). Saplings of the plants were purchased from the licensed supplier of certified crops and ornamental plants, the Polish Vegetable Seed Farming and Nursery enterprise “PNOS” and cultivated in an open field. Samples were collected from plants at the full-flowing stage and air-dried at room temperature.

### 4.2. Extraction

Samples of dried plant material (three replicates each) were powdered, weighed and extracted in a Soxhlet apparatus for 8 h with diethyl ether. Diethyl ether was chosen due to a proven high extraction yield of triterpenoids and phytosterols from dried plant material (approx. 96% [32]). The obtained extracts were evaporated to dryness at 40 °C under reduced pressure on a rotary evaporator.

### 4.3. Fractionation of Diethyl Ether Extracts

Evaporated diethyl ether extracts were fractionated by adsorption preparative TLC on 20 cm × 20 cm glass plates, manually coated with silica gel 60H (Merck, Darmstadt, Germany) in the solvent system chloroform: methanol 97:3 (*v*/*v*). Two fractions were obtained: (i) free steroids and neutral triterpenoids (Rf of 0.3–0.9), and (ii) free triterpenoid acids (Rf 0.2–0.3), as described earlier [32,62]. The individual fractions were localized and plated by comparison with standards of oleanolic acid, sitosterol and α-amyrin, visualized by spraying the relevant part of the plate with 50% H_2_SO_4_ followed by heating with a hot-air stream. Fractions were eluted from the gel in diethyl ether. The fraction of free steroids and neutral triterpenoids was directly analyzed without derivatization using GC–MS (Agilent Technologies 7890A) while the fraction of triterpenoid acids was methylated with diazomethane [32,33].

### 4.4. Derivatization of Triterpenoid Acids

Nitrosomethylurea (2.06 g) was added to a mixture of 20 mL of diethyl ether and 6 mL of 50% aqueous KOH. The organic layer was separated and used to dissolve the samples of triterpenoid acids (2 mL per sample). The reaction was held at 2 °C for 24 h.

### 4.5. Identification and Quantification of Steroids by Gas Chromatography-Mass Spectrometry

An Agilent Technologies 7890A gas chromatograph (GC–MS) (Perlan Technologies, Warszawa, Poland) equipped with a 5975C mass selective detector was used for qualitative and quantitative analyses. The column used was a 30 m × 0.25 mm i.d., 0.25-μm, HP-5MS UI (Agilent Technologies, Santa Clara, CA, USA). Helium was used as the carrier gas at a flow rate of 1 mL/min. Analyzed samples were dissolved in the mixture of diethyl ether:methanol (5:1, *v*/*v*) and applied (in a volume of 1–4 μL) using a 1:10 split injection. The separation was made with the temperature program: an initial temperature of 160 °C was held for 2 min, then increased to 280 °C at 5 °C/min, and the final temperature of 280 °C was held for a further 44 min. The other employed parameters were as follows: inlet and FID (flame ionization detector) temperature 290 °C; MS transfer line temperature 275 °C; quadrupole temperature 150 °C; ion source temperature 230 °C; EI 70 eV; *m*/*z* range 33–500; FID gas (H2) flow 30 mL·min^−1^ (hydrogen generator HydroGen PH300, Peak Scientific, Inchinnan, UK); and airflow 400 mL·min^−1^. Individual compounds were identified by comparing their mass spectra with library data from Wiley 9th ED. and NIST 2008 Lib. SW Version 2010, or previously reported data, and by comparison of their retention times and corresponding mass spectra with those of authentic standards, where available. Quantitation was performed using an external standard method based on calibration curves determined for authentic standards of sitosterol, α-amyrin and oleanolic acid methyl ester, applied for each class of compounds (i.e., steroids, neutral triterpenoids and triterpenoid acids, respectively) as described earlier [32,33,62].

### 4.6. Statistical Analysis of Data

Data are presented as the means ± standard deviation of three independent samples analyzed in triplicate. The data were subjected to one-way analysis of variance (ANOVA), and the differences between means were evaluated using Duncan’s multiple-range test. Statistical significance was considered to be obtained at *p* < 0.05.

The statistical comparison of the content of each individual compound was made among all analyzed plant species, separately in flowers/inflorescences and in leaves; and the statistically significant differences of this inter-species comparison were expressed in capital letters (A–F). The intra-species statistically significant differences between flowers/inflorescences and leaves of the same plant (except for *S. nigra*, for which the comparison of the wild and ornamental cultivars was performed) were expressed in lowercase (a, b).

## 5. Conclusions

Flowers have recently gained great attention owing to their health-supporting potential due to their particular richness in bioactive and nutraceutical compounds; and hence the possibility of application in modern nutraceuticals, functional foods, dietary supplements and cosmetic products. The obtained results revealed that flowers and inflorescences of various medicinal plants differed markedly in steroid and triterpenoid composition and content. Steroids were the most abundant in *P. vulgaris* and *V. minor* flowers (0.11% and 0.08% d.w., respectively). The richest source of the neutral triterpenoids (alcohols, ketones, aldehydes) were *V. minor* flowers (0.54% d.w.) and *C. laevigata* inflorescences (0.16% d.w.), whereas the most abundant source of triterpenoid acids was wild and ornamental *S. nigra* inflorescences (1.28% and 0.87% d.w). The composition of triterpenoids was the most complex in *C. laevigata, R. rugosa, S. nigra* and *V. minor,* whereas the simplest in *P. vulgaris* (only oleanolic and ursolic acids). The present results revealed that flowers and inflorescences of some medicinal plants less characterized for triterpenoid content can be valuable sources of these compounds; moreover, the data concerning the occurrence of bioactive triterpenoids in herbal material can widen the spectrum of its potential applications.

## Figures and Tables

**Table 1:** The content of steroids and triterpenoids in inflorescences and leaves of *Berberis vulgaris*.

Compound/Category	Inflorescences	Leaves
	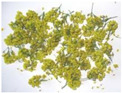	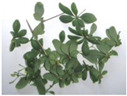
	**µg/g d.w.**	
*Steroids*:		
Campesterol	31.50 ± 2.49 a, A	46.65 ± 4.26 b, A
Stigmasterol	11.71 ± 0.96 a, A	11.77 ± 0.57 a, A
Sitosterol	55.45 ± 4.76 a, A	285.56 ± 19.38 b, A
24-methylenecycloartanol	18.51 ± 1.37 a, A	26.77 ± 1.88 b, A
Tremulone	38.56 ± 2.84 a, A	177.05 ± 15.18 b, A
*Sum of steroids*	*155.73*	*547.82*
*Triterpenoid acids:*		
Oleanolic acid	45.82 ± 3.48 a, A	38.92 ± 3.22 a, A
Ursolic acid	76.82 ± 5.69 a, A	79.68 ± 6.65 a, A
Olean-2,12-dien-28-oic acid	3.28 ± 0.40 A	n.d.
Ursa-2,12-dien-28-oic acid	10.94 ± 0.88 A	n.d.
3-oxo-oleanolic acid	1.89 ± 0.02 A	n.d.
3-oxo-ursolic acid	3.62 ± 0.24 A	n.d.
*Sum of triterpenoid acids*	*142.37*	*118.60*
Total	298.10	666.42

Results are referenced to inflorescences and leaf dry weight and expressed as the mean ± SD of three samples. The statistical difference of the content of each individual compound was analyzed among all analyzed plant species (significance expressed in capital letters) as well as between flowers/inflorescences and leaves of the same plant (expressed in lowercase), as described in Section 4.6. Results not sharing a common letter are significantly different (*p* < 0.05). n.d.—not detected.

**Table 2:** The content of steroids and triterpenoids in inflorescences and leaves of *Crategus laevigata*.

Compound/Category	Flowers	Leaves
	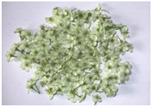	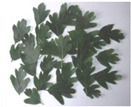
	**µg/g d.w.**	
*Steroids*:		
Sitosterol	62.80 ± 5.64 a, A	118.92 ± 10.06 b, B
Tremulone	61.75 ± 5.97 a, B	55.64 ± 5.02 b, B
*Sum of steroids*	*124.55*	*174.56*
*neutral triterpenoids:*		
α-amyrine/lupeol	1346.22 ± 108.94 a, A	34.87 ± 3.50 b, A
α-amyrenone	n.d.	32.60 ± 3.09 a, A
β-amyrine	94.81 ± 10.20 a, A	25.98 ± 2.62 b, A
Erythrodiol	n.d.	34.51 ± 3.35 a, A
Germanicol	n.d.	56.98 ± 5.26 a, A
Uvaol	n.d.	37.14 ± 3.58 a, A
Oleanolic aldehyde	17.62 ± 1.68 A	n.d.
Ursolic aldehyde	186.80 ± 20.45 A	n.d.
*Sum of neutral triterpenoids*	*1645.45*	*222.08*
*Triterpenoid acids:*		
Oleanolic acid	357.57 ± 30.15 a, B	1277.52 ± 118.84 b, B
Betulinic acid	21.09 ± 2.11 a, A	86.66 ± 8.50 b, A
Ursolic acid	769.14 ± 68.64 a, B	4506.87 ± 401.11 b, B
Olean-2,12-dien-28-oic acid	13.96 ± 1.42 a, B	27.80 ± 3.12 b, A
Ursa-2,12-dien-28-oic acid	47.84 ± 4.06 a, B	102.55 ± 11.70 b, A
3-oxo-oleanolic acid	23.56 ± 2.22 a, B	n.d.
3-oxo-ursolic acid	27.94 ± 2.86 a, B	n.d.
*Sum of triterpenoid acids*	*1261.10*	*6001.40*
Total	3031.10	6398.04

Results are referenced to inflorescences and leaf dry weight and expressed as the mean ± SD of three samples. Results not sharing a common letter are significantly different (*p* < 0.05). n.d.—not detected.

**Table 3:** The content of steroids and triterpenoids in flowers and leaves of *Pulsatilla vulgaris*.

Compound/Category	Flowers	Leaves
	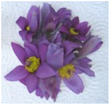	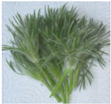
	**µg/g d.w.**	
*Steroids:*		
Campesterol	160.40 ± 14.86 a, B	25.72 ± 2.60 b, B
Isofucosterol	57.39 ± 5.40 a, A	n.d.
Stigmasterol	171.44 ± 15.26 a, B	22.06 ± 2.28 b, B
Sitosterol	645.31 ± 65.05 a, B	101.19 ± 12.55 b, B
Sitostenone	73.68 ± 7.18 a, A	27.99 ± 2.31 b, A
Tremulone	28.71 ± 3.05 a, C	63.22 ± 6.08 b, A
*Sum of steroids*	*1136.93*	*240.18*
*Triterpenoid acids*		
Oleanolic acid	20.58 ± 1.94 a, C	22.32 ± 2.46 a, C
Ursolic acid	5.18 ± 0.46 a, C	6.26 ± 0.58 a, C
*Sum of triterpenoid acids*	*25.76*	*28.58*
Total	1162.69	268.76

Results are referenced to flower and leaf dry weight and expressed as the mean ± SD of three samples. Results not sharing a common letter are significantly different (*p* < 0.05). n.d.—not detected.

**Table 4:** The content of steroids and triterpenoids in flowers and leaves of *Rosa rugosa*.

Compound/Category	Flowers	Leaves
	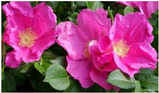	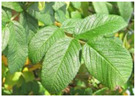
	**µg/g d.w.**	
*Steroids:*		
Campesterol	16.62 ± 1.58 a, C	55.74 ± 6.01 b, A
Isofucosterol	33.78 ± 4.02 a, B	60.12 ± 5.94 b, A
Stigmasterol	8.12 ± 0.98 a, C	39.93 ± 4.05 b, C
Sitosterol	329.30 ± 20.71 a, C	410.41 ± 29.95 b, C
24-methylenecycloartanol	21.44 ± 2.66 a, A	55.26 ± 5.72 b, B
Obtusifoliol	18.17 ± 1.85 a, A	28.79 ± 3.11 b, A
Sitostenone	6.39 ± 0.62 a, B	18.62 ± 2.04 b, B
Tremulone	22.61 ± 2.30 a, C	25.50 ± 2.63 a, C
*Sum of steroids*	*456.43*	*694.37*
*Neutral triterpenoids*:		
α-amyrine/lupeol	284.78 ± 30.04 a, B	158.92 ± 16.88 b, B
α-amyrenone	32.54 ± 3.30 a, A	22.15 ± 2.31 b, B
β-amyrine	92.04 ± 8.98 a, A	66.32 ± 6.74 b, B
Erythrodiol	48.12 ± 5.04 a, A	n.d.
Uvaol	142.58 ± 15.66 a, A	n.d.
Oleanolic aldehyde	12.88 ± 1.31 a, B	28.34 ± 3.02 b, A
Ursolic aldehyde	45.13 ± 4.48 a, B	45.86 ± 4.23 a, A
*Sum of neutral triterpenoids*	*530.07*	*321.59*
*Triterpenoid acids*:		
Betulinic acid	35.75 ± 3.60 a, B	10.82 ± 1.07 b, B
Corosolic acid	138.99 ± 14.55 a, A	147.06 ± 15.83 a, A
Maslinic acid	36.04 ± 3.46 a, A	14.23 ± 1.57 b, A
Pomolic acid	48.33 ± 5.01 a, A	102.93 ± 10.55 b, A
Oleanolic acid	250.41 ± 26.13 a, D	152.34 ± 14.08 b, D
Ursolic acid	490.82 ± 50.04 a, D	362.20 ± 35.16 b, D
Olean-2,12-dien-28-oic acid	n.d.	20.62 ± 2.44 a, A
Ursa-2,12-dien-28-oic acid	n.d.	30.77 ± 3.05 a, B
3-oxo-oleanolic acid	n.d.	22.71 ± 2.33 a, A
3-oxo-ursolic acid	n.d.	61.74 ± 6.28 a, A
*Sum of triterpenoid acids*	*1000.34*	*925.42*
Total	1986.84	1941.38

Results are referenced to flower and leaf dry weight and expressed as the mean ± SD of three samples. Results not sharing a common letter are significantly different (*p* < 0.05). n.d.—not detected.

**Table 5:** The content of steroids and triterpenoids in the flowers of wild *S. nigra* and ornamental *S. nigra* f. *porphyrophylla* Black lace “Eva”.

Compound/Category	Wild	Ornamental
	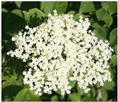	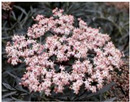
	**µg/g d.w.**	
*Steroids*:		
Campesterol	90.58 ± 8.69 a, D	71.60 ± 6.23 b, E
Isofucosterol	6.84 ± 0.58 a, C	8.21 ± 0.79 a, C
Sitosterol	431.16 ± 51.73 a, D	363.84 ± 22.67 b, C
Sitostanol	7.23 ± 0.58 a, A	6.11 ± 0.56 a, A
24-methylenecycloartanol	72.23 ± 6.57 a, B	51.50 ± 3.92 b, C
Tremulone	94.24 ± 10.36 a, D	60.07 ± 5.89 b, B
*Sum of steroids*	*702.28*	*561.33*
*Neutral triterpenoids:*		
α-amyrine/lupeol	99.24 ± 5.76 a, C	77.14 ± 5.25 b, D
α-amyrenone	42.04 ± 3.49 a, B	21.46 ± 1.95 b, C
β-amyrine	42.42 ± 3.08 a, B	28.41 ± 2.61 b, C
Taraxasterol	3.27 ± 0.21 a, A	3.52 ± 0.29 a, A
Erythrodiol	4.58 ± 0.48 a, B	3.31 ± 0.31 a, B
Germanicol	15.61 ± 1.23 a, A	7.88 ± 0.66 b, B
Uvaol	20.93 ± 1.75 a, B	17.30 ± 1.29 a, B
Oleanolic aldehyde	1.50 ± 0.13 a, C	12.95 ± 0.89 b, B
Ursolic aldehyde	144.87 ± 9.28 a, A	93.89 ± 5.45 b, C
*Sum of neutral triterpenoids*	*374.46*	*265.86*
*Triterpenoid acids:*		
Oleanolic acid	3686.88 ± 365.00 a, E	2137.63 ± 198.79 b, F
Ursolic acid	7684.76 ± 423.20 a, E	5840.09 ± 443.85 b, F
Olean-2,12-dien-28-oic acid	343.54 ± 28.17 a, C	157.40 ± 15.32 b, D
Ursa-2,12-dien-28-oic acid	989.00 ± 72.20 a, C	457.37 ± 36.13 b, D
3-oxo-oleanolic acid	46.62 ± 3.21 a, C	25.42 ± 2.41 b, B
3-oxo-ursolic acid	84.65 ± 8.63 a, C	67.85 ± 4.82 b, D
Oleanolic acid methyl ester	25.41 ± 1.91 a, A	6.53 ± 0.38 b, B
Ursolic acid methyl ester	3.42 ± 0.28 a, A	5.77 ± 0.42 b, B
*Sum of triterpenoid acids*	12,864.28	8698.06
Total	13,941.02	9525.25

Results are referenced to inflorescences and leaf dry weight and expressed as the mean ± SD of three samples. Results not sharing a common letter are significantly different (*p* < 0.05).

**Table 6:** The content of steroids and triterpenoids in flowers of and leaves of *Vinca minor*.

Compound/Category	Flowers	Leaves
	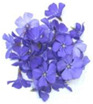	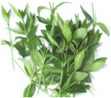
	**µg/g d.w.**	
*Steroids*:		
Campesterol	89.53 ± 9.75 a, D	151.19 ± 14.80 b, C
Stigmasterol	40.42 ± 4.14 a, D	61.08 ± 6.22 b, D
Sitosterol	280.13 ± 26.05 a, C	362.63 ± 3.75 b, C
24-methylenecycloartanol	321.16 ± 30.98 a, D	n.d.
Sitostenone	107.97 ± 9.86 a, C	95.03 ± 9.55 a, C
Tremulone	n.d.	93.47 ± 9.20 a, D
*Sum of steroids*	*839.21*	*763.40*
*Neutral triterpenoids:*		
α-amyrine/lupeol	2999.49 ± 280.15 a, E	400.75 ± 38.01 b, C
α-amyrenone	99.22 ± 8.86 a, D	n.d.
β-amyrine	55.65 ± 5.20 a, D	214.47 ± 20.13 b, C
Betulin	n.d.	258.25 ± 24.90 a, A
Lupeol acetate	2263.36 ± 203.72 a, A	n.d.
Oleanolic aldehyde	n.d.	50.37 ± 4.95 a, B
Ursolic aldehyde	n.d.	281.06 ± 25.88 a, B
*Sum of neutral triterpenoids*	*5417.72*	*1204.90*
*Triterpenoid acids:*		
Oleanolic acid	219.76 ± 20.04 a, D	26.78 ± 2.76 b, C
Oleanolic acid acetate	106.51 ± 9.35 a, A	n.d.
Ursolic acid	654.55 ± 63.01 a, B	83.05 ± 8.40 b, A
*Sum of triterpenoid acids*	*980.82*	*109.83*
Total	7237.75	2028.13

Results are referenced to flower and leaf dry weight and expressed as the mean ± SD of three samples. Results not sharing a common letter are significantly different (*p* < 0.05). n.d.—not detected.

## Data Availability

The data presented in this study are available in the article and Supplementary Materials.

## Supplementary Materials

The following supporting information can be downloaded at https://www.mdpi.com/2223-7747/12/9/1838/s1, Table S1. GC-MS data (retention times and characteristic ions of mass spectra) of identified steroids and triterpenoids.
