# Correction: Edorh Tossa et al. Flowers and Inflorescences of Selected Medicinal Plants as a Source of Triterpenoids and Phytosterols. *Plants* 2023, *12*, 1838

**DOI:** 10.3390/plants12213697

**Published:** 2023-10-26

**Authors:** Pauline Edorh Tossa, Morgan Belorgey, Soyol Dashbaldan, Cezary Pączkowski, Anna Szakiel

**Affiliations:** 1 Clermont Auvergne Institut National Polytechnique, SIGMA Clermont, Campus des Cézeaux CS 20265, 63178 Aubière, France; p.edorhtossa@gmail.com; 2 Faculté de Pharmacie, Université Clermont Auvergne, 28 Place Henri Dunant, BP 38, 63001 Clermont-Ferrand, France; morgan.belorgey@hotmail.com; 3 School of Industrial Technology, Mongolian University of Science and Technology, 8th Khoroo, Baga Toiruu 34, Sukhbaatar District, Ulaanbaatar 14191, Mongolia; soyol_d@must.edu.mn; 4 Department of Plant Biochemistry, Faculty of Biology, University of Warsaw, 1 Miecznikowa Street, 02-096 Warsaw, Poland; c.paczkowski@uw.edu.pl

In the original publication [1], there was a mistake in Figure 4, which showed the selected plants in their flowering stage (the photographs were taken by Anna Szakiel). The mistake was the replacement of the first two photographs. The corrected Figure 4 appears below.

The authors state that the scientific conclusions are unaffected. This correction was approved by the Academic Editor. The original publication has also been updated.

## Figures and Tables

**Figure 4:**
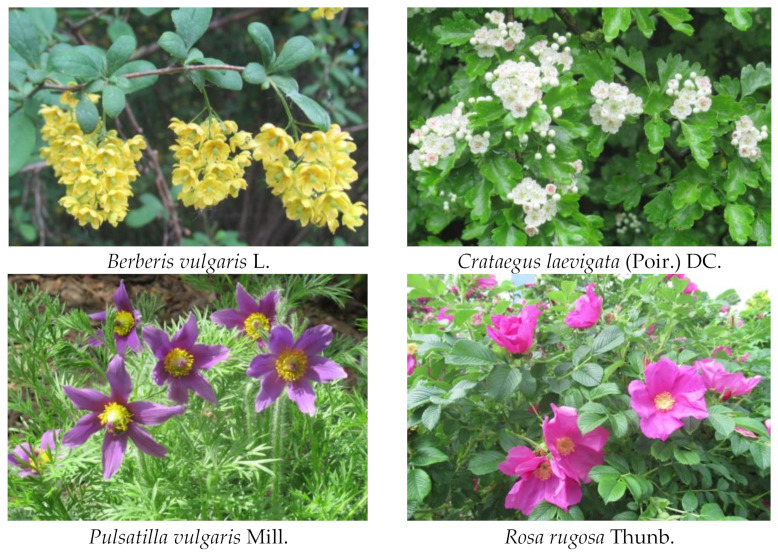

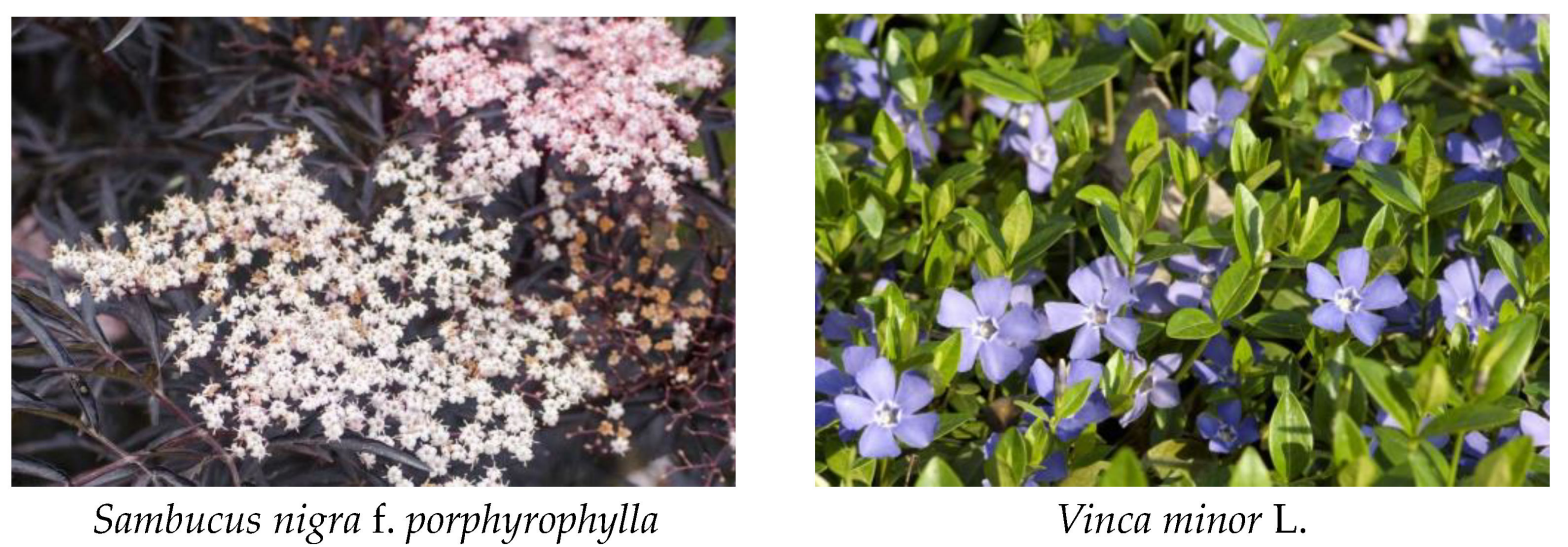
Selected plants in their flowering stage (the photographs taken by Anna Szakiel).

## References

[1] Edorh Tossa P., Belorgey M., Dashbaldan S., Pączkowski C., Szakiel A. (2023). Flowers and Inflorescences of Selected Medicinal Plants as a Source of Triterpenoids and Phytosterols. Plants.

