# Supplementary material for: Flowers and Inflorescences of Selected Medicinal Plants as a Source of Triterpenoids and Phytosterols
Source: Plants (Basel). 2023 Apr 29;12(9):1838. doi: 10.3390/plants12091838 (PMC10181404; doi:10.3390/plants12091838)
Supplement: Supplementary file 1 [file plants-12-01838-s001.zip › plants-2316017-supplementary.pdf]

Flowers and inflorescences of selected medicinal plants as a source of triterpenoids and phytosterols

Table S1. Retention times and characteristic ions of mass spectra of identified steroids and triterpenoids

Retention time	Compound	Mass spectrum *m*/*z* (relative intensity)
34.6	Campesterol	400 (30), 107 (51), 105 (55), 95 (49), 83 (45), 81 (64), 71 (62), 57 (77), 55 (77), 43 (100), 41 (52)
35.5	Stigmasterol	412 (36), 145 (64), 107 (52), 95 (100), 83 (66), 81 (90), 78 (60), 69 (67), 67 (85), 55 (69)
36.0	Obtusifoliol	426 (41), 412 (34), 411 (100), 245 (24), 109 (24), 95 (30), 81 (21), 69 (34), 55 (36), 41 (20)
37.5	Sitosterol	414 (29), 145 (54), 107 (59), 105 (60), 95 (54), 91 (49), 81 (57), 57 (68), 55 (70), 43 (100), 41 (44)
37.6	Sitostanol	416 (31), 215 (82), 109 (58), 107 (83), 95 (81), 93 (64), 81 (84), 69 (60), 57 (64), 55 (81), 43 (100)
37.8	Isofucosterol	412 (5), 314 (100), 105 (47), 95 (50), 91 (42), 83 (40), 81 (51), 69 (61), 55 (96), 43 (49)
38.2	Germanicol	426 (1), 204 (100), 177 (85), 189 (75), 95 (58), 55 (46), 205 (44), 81 (42), 109 (40), 69 (39), 107 (37)
38.5	β-Amyrin	426 (27), 219 (18), 218 (100), 203 (49), 189 (17), 135 (11), 109 (13), 105 (12), 95 (15), 81 (18), 69 (14)
39.5	α-Amyrenone	424 (12), 219 (19), 218 (100), 203 (24), 189 (16), 135 (19), 133 (18), 122 (18), 119 (17), 95 (16), 55 (18)
39.4	α-Amyrin/	426 (4), 218 (100), 203 (20), 189 (36), 135 (35), 121 (32), 109 (32), 107 34), 95 (40), 81 (33), 55 (31)
	Lupeol	426 (18), 207 (67), 189 (90), 135 (83), 121 (80) 109 (85), 121 (80), 95 (100), 93 (87), 81 (86),
40.7	Tremulone (stigmasta-3,5-dien-7-one)	410 (32), 187 (27), 174 (100), 161 (37), 159 (26), 91 (28), 57 (28), 55 (37), 43 (44), 41 (28)
41.4	24-methylene-cycloartanol	440 (5), 121 (60), 119 (55), 109 (62), 107 (76), 105 (57), 95 (98), 93 (64), 81 (72), 69 (99), 55 (100)
41.9	Sitostenone	440 (5), 121 (60), 119 (55), 109 (62), 107 (76), 105 (57), 95 (98), 93 (64), 81 (72), 69 (99), 55 (100)
43.5	Taraxasterol	426 (14), 207 (57), 189 (100), 135 (51), 121 (74), 109 (57), 107 (62), 95 (70), 93 (47), 81 (48), 67 (43)

44.0	Lupeol acetate	468 (8), 189 (100), 135 (63), 121 (76), 109 (71), 107 (78), 95 (77), 93 (80), 81 (68), 69 (53)
48.0	Oleanolic aldehyde	440 (2), 232 (28), 207 (20), 204 (39), 203 (100), 189 (29), 105 (18), 81 (19), 69 (20), 55 (29)
50.9	Ursolic aldehyde	440 (1), 207 (26), 204 (23), 203 (100), 133 (42), 119 (18), 105 (18), 95 (18), 81 (18), 55 (18), 43 (20)
52.7	Erythrodiol	442 (1), 204 (17), 203 (100), 133 (7), 119 (9), 105 (8), 95 (9), 93 (8), 81 (8), 69 (9), 55 (8)
55.7	Uvaol	442 (1), 207 (13), 204 (17), 203 (100), 133 (33), 119 (13), 105 (11), 95 (12), 81 (10), 69 (10), 55 (11)
57.2	Betulin	442 (8), 203 (100), 189 (77), 133 (66), 121 (55), 107 (57), 105 (49), 95 (56), 93 (54), 81 (67)
Acids analyzed after methylation:		
42.0	Olean-2,12-dien-28-oic acid methyl ester	452(11), 425 (9), 263 (11), 262 (61), 221 (14), 203 (100), 190 (15), 189 (22), 133 (14), 119 (12)
44.7	Ursa-2,12-dien-28-oic acid methyl ester	452 (12), 425 (9), 263 (20), 262 (100),221 (27), 203 (79), 190 (18), 189 (27), 133 (58), 119 (23)
46.0	3-Oxo-olean-12-en-28-oic acid methyl ester	468 (6), 262 (32), 204 (17), 203 (100), 202 (21), 189 (29),133 (17), 119 (14), 105 (12), 55 (12)
46.7	Oleanolic acid methyl ester	470 (1), 262 (48), 207 (13), 204 (16), 203 (100), 202 (21), 189 (22), 133 (17), 119 (13), 105 (14)
47.1	Betulinic acid methyl ester	470 (5), 207 (41), 203 (38), 189 (100), 175 (40), 119 (41), 107 (38), 105 (37), 95 (37), 93 (38)
47.5	3-Oxo-urs-12-en-28-oic acid methyl ester	468 (3), 263 (21), 262 (96), 249 (20), 204 (17), 203 (100), 189 (29), 133 (79), 119 (30), 105 (19)
49.7	Ursolic acid methyl ester	470 (1), 263 (20), 262 (100), 207 (32), 203 (93), 189 (29), 133 (76), 119 (34), 105 (21), 95 (18)
61.0	Maslinic acid methyl ester	486 (2), 263 (10), 262 (53), 204 (17), 203 (100), 202 (20), 189 (20), 133 (16), 119 (13), 105 (12), 69 (10)
63.0	Corosolic acid methyl ester	486 (1), 263 (15), 262 (74), 204 (17), 203 (100), 202 (22), 189 (21), 119 (18), 105 (14), 55 (12)
73.0	Pomolic acid methyl ester	486 (3) 263 (12), 263 (55), 204 (16), 203 (100), 202 (20), 189 (17), 119 (12), 105 (10), 75 (14)
